# Leveraging artificial intelligence for evidence‐based recommendations in uterine fibroid therapy: Addressing the unmet need in German healthcare—A clinical trial

**DOI:** 10.1002/ijgo.70407

**Published:** 2025-07-29

**Authors:** Iason Psilopatis, Laura Lotz, Nadezda Sipulina, Felix Heindl, Georgia Levidou, Julius Emons

**Affiliations:** ^1^ Department of Gynecology and Obstetrics Universitätsspital Basel Basel Switzerland; ^2^ Department of Gynecology and Obstetrics Universitätsklinikum Erlangen, Comprehensive Cancer Center Erlangen‐EMN (CCC ER‐EMN), Friedrich‐Alexander‐Universität Erlangen‐Nürnberg (FAU) Erlangen Germany; ^3^ Department of Pathology, Nuremberg Clinic Paracelsus Medical University Nuremberg Germany

**Keywords:** artificial intelligence, fibroid, guideline, treatment, uterus

## Abstract

**Objective:**

To evaluate the potential of an artificial intelligence (AI)‐driven large language model, ChatGPT 4.0, to provide personalized, evidence‐based treatment recommendations for uterine fibroids.

**Methods:**

ChatGPT 4.0 was trained using evidence‐based data from Uptodate and German medical literature. The algorithm generated individualized recommendations based on clinical characteristics and patient preferences. Usability and quality were assessed through questionnaires completed by 40 gynecologists and 45 women with fibroids.

**Results:**

Most gynecologists found the algorithm user‐friendly and comprehensive, with 15 expressing a willingness to integrate it into practice and 24 acknowledging its potential to enhance healthcare efficiency. Although only half believed it would improve patient outcomes, the tool was generally well received. Patients found the algorithm easy to understand and helpful for exploring treatment options, with the majority feeling it empowered informed discussions with their healthcare providers. A minority expressed dissatisfaction with usability or helpfulness.

**Conclusion:**

ChatGPT 4.0 offers a promising AI‐driven tool for personalized fibroid management in the absence of formal guidelines. Although not a substitute for official recommendations, it could support clinical decision making and enhance patient education. Further integration with standardized guidelines and prospective trials is needed to optimize its clinical utility.

## 1 INTRODUCTION

Uterine fibroids, also known as leiomyomas or myomas, are benign tumors originating from smooth muscle cells within the uterine wall.^1^ They represent one of the most common gynecologic conditions affecting women of reproductive age.^2^ Many women with uterine fibroids remain asymptomatic, but these tumors can manifest with a diverse array of symptoms depending on their size, location, and number.^3^

Symptoms of uterine fibroids can vary widely, ranging from heavy menstrual bleeding (menorrhagia) and pelvic pain to pressure symptoms such as urinary frequency, constipation, and pelvic pressure. Furthermore, fibroids located near the uterine cavity (submucosal) may contribute to infertility and recurrent miscarriages. The clinical presentation of uterine fibroids is highly heterogeneous, necessitating individualized approaches to management.^4^

In Germany, specialized centers offer (second opinion) consultation programs for patients with uterine fibroids, providing expert opinions on the diverse therapeutic options available. These options encompass a spectrum of interventions, ranging from conservative measures such as watchful waiting and medical management to surgical (myomectomy or hysterectomy) and interventional procedures including uterine artery embolization, high‐intensity focused ultrasound ablation and transcervical radiofrequency ablation.^5^, ^6^, ^7^, ^8^, ^9^ However, despite the broad expertise available, there is still a notable absence of an official national guideline for the treatment of uterine fibroids in Germany.

The lack of a standardized guideline underscores the need for innovative solutions to support clinicians and patients in decision making processes regarding uterine fibroid therapy. Artificial intelligence (AI) technologies, such as ChatGPT, offer a promising avenue for addressing this unmet need. By leveraging natural language processing and machine learning algorithms, ChatGPT can help analyze vast volumes of medical literature and real‐world data to generate feasible evidence‐based recommendations tailored to individual patient profiles.^10^, ^11^, ^12^, ^13^, ^14^

In this paper, we explore the potential of the AI‐driven platform ChatGPT 4.0 for uterine fibroid therapy in Germany. Through a research study, we aim to evaluate the efficacy, accuracy, and usability of ChatGPT‐generated recommendations. By facilitating informed decision making and personalized care, we seek to enhance the management of uterine fibroids and improve patient outcomes in the German healthcare system.

## 2 MATERIALS AND METHODS

### 2.1 Data collection

We collected relevant information from two distinct sources to train ChatGPT for providing recommendations on uterine fibroid therapy: Uptodate and the German medical literature (*Deutsches Ärzteblatt*).

We obtained evidence‐based recommendations on uterine fibroid therapy from the leading evidence‐based clinical resource Uptodate. This source provided comprehensive recommendations, including therapeutic options, eligibility criteria, and clinical outcomes associated with various interventions.^15^

Additionally, we sourced information from the two seminal German articles published in the official Journal of the German Medical Society *Deutsches Ärzteblatt*. These articles offered insights into the unique considerations and therapeutic approaches relevant to uterine fibroid management in the German healthcare context.^16^, ^17^

### 2.2 Data preparation

We meticulously curated the information obtained from Uptodate and the German medical literature to ensure relevance and accuracy. This involved extracting key recommendations, therapeutic modalities, eligibility criteria, and clinical outcomes pertaining to uterine fibroid therapy.

### 2.3 Training ChatGPT

ChatGPT 4.0 was trained using the curated data sets obtained from Uptodate and the German medical literature separately. Each data set served as a distinct knowledge source, reflecting international best practices and local expertise in uterine fibroid therapy. The relevant abstracts concerning diagnostic and therapeutic approaches were manually extracted and subsequently provided in plain‐text format to ChatGPT in the English language by a human expert with proficiency in both the English and the German language in order to ensure accurate model training.

### 2.4 Algorithm development

Following the training phase, we tasked ChatGPT with synthesizing the information from both Uptodate and the German medical literature to generate evidence‐based recommendations for uterine fibroid therapy. The algorithm developed by ChatGPT aimed to identify the most suitable therapeutic options for individual patients based on their clinical characteristics, preferences, and eligibility criteria outlined in the provided information (Data S1). The English version of the algorithm was translated into German by a human expert with proficiency in both the English and the German language.

### 2.5 Algorithm evaluation

Two distinct questionnaires were designed with a view to giving both physicians and patients the chance to evaluate the developed algorithm and to provide feedback on the usability and quality of this tool (Data S2). The questionnaires were developed in accordance with the satisfaction questionnaires employed by the Department of Gynecology of the University Hospital of Erlangen for the evaluation of therapeutic approaches in diverse gynecologic conditions. Most questions have five response items (Likert scale model) for quantitative assessment. The English versions of the questionnaires were translated into German by a human expert with proficiency in both the English and the German language. The questionnaires were administered to patients and physicians between September and December 2024.

### 2.6 Ethical considerations

This study adhered to ethical guidelines governing the use of artificial intelligence in healthcare research, ensuring patient privacy, confidentiality, and informed consent. The study received ethical approval from the Ethics Committee of the Friedrich Alexander University of Erlangen (approval number: 24‐226‐B).

### 2.7 Statistical analysis

During descriptive analysis, the parameters were used without categorization in subgroups. During statistical analysis, we categorized each parameter in two subgroups, i.e. regarding low or high levels of satisfaction, while evaluating the algorithm. In case of a neutral answer, this observation was not classified as a positive evaluation of the algorithm. The level of familiarity of using AI in medicine (evaluated on a scale from 0 to 10) was classified into three categories, as follows: low (0–3 points), moderated (4–6 points), and high (6–10 points). Missing values were not included in the analysis. Statistical analysis was performed using Fisher exact test, as appropriate. A *P*‐value less than 0.05 was considered statistically significant. A value of *P* between 0.05 and 0.10 was considered of marginal significance. Statistical analysis was performed using the statistical package STATA 11.0/SE for Windows (StataCorp, College Station, TX, USA).

## 3 RESULTS

The first part of the algorithm focuses on the different factors on which the right decision as to which therapy might be suitable for each patient depends. These factors ranged from clinical symptoms and fibroid characteristics to previous medical conditions and the wishes of the patient. The second part of the algorithm incorporated the inclusion and exclusion criteria for each possible therapeutic approach, while the third part of the algorithm suggested first‐line and second‐line therapeutic approaches for uterine fibroids (Data S1).

In order to assess the developed algorithm and examine the tool's quality and usability, a total of 40 consulting physicians and 45 patients with fibroids completed two distinct evaluation questionnaires between September and December 2024 (Data S2).

### 3.1 Physicians' responses

All physicians were gynecologists working in the Department of Gynecology and Obstetrics, University Hospital Erlangen, Germany. Most of the physicians consulted patients with fibroids in their daily clinical routine on a regular basis (92.5%), were not very (47.4%) or moderately (42.1%) familiar with the use of AI in medicine, and were satisfied with, or at least neutral, concerning the user‐friendliness and quality of the AI‐generated algorithm for myoma treatment. No gynecologists found the algorithm difficult to understand and use in the clinical routine and rated its quality as low (except for three physicians). The majority of the physicians found the algorithm (partially) comprehensive in terms of the different aspects of fibroid treatment, with, however, only half believing that the algorithm would help to improve patient outcomes by providing personalized treatment options. Fifteen physicians (37.5%) would (very) likely integrate this algorithm into the clinical routine for the treatment of fibroid patients, 17 physicians (42.5%) would recommend this algorithm to their colleagues, while 24 physicians (60%) believed that using this algorithm could lead to more efficient use of healthcare resources in fibroid treatment. The questions, *Do you find the algorithm easy to understand and applicable in your daily clinical routine?* and *Overall, how satisfied are you with the user‐friendliness and quality of the AI‐generated myoma treatment algorithm?* were the ones to receive the highest percentage of positive replies by the physicians questioned, whereas the questions *Do you think the algorithm will help improve patient outcomes by providing personalized treatment options?* and *How likely is it that you will integrate this algorithm into your clinical routine for the treatment of fibroid patients?* were the ones to receive the highest percentage of negative replies by the clinicians (Table 1). Interestingly, there was no significant association between the familiarity of clinicians using AI in medicine and the evaluation of the algorithm in the use of treatment of fibroids (Fisher exact test *P* < 0.10 for all associations; evaluation of algorithm easiness *P* = 0.751, algorithm quality *P* = 0.723, algorithm comprehesivenes *P* = 0.223, improvement of clinical outcome *P* = 0.653, intergration into routine practice *P* = 0.898, recommendation to colleagues *P* = 0.567, promotion of efficient use of healthcare sources *P* = 0.567, overall user‐friendliness and quality *P* = 0.340). The same applied to the frequency of dealing with fibroids in routine practice and the evaluation of the algorithm in the use of treatment of fibroids (Fisher exact test *P* < 0.10 for all associations).

**TABLE 1:** Doctors‘ responses to the study questions regarding the algorithm.

How often do you see patients with fibroids in your daily clinical routine?	Frequency
Very often	7
Often	19
Sometimes	11
Seldomly	3
Never	0
Total	40
Invalid	0
Total	40

	Statistical analysis
Mean	2.25
Std. Deviation	0.84
Minimum	1
Maximum	4

Do you find the algorithm easy to understand and applicable in your daily clinical routine?	Frequency
Very easy	6
Easy	23
Neutral	10
Difficult	0
Very difficult	0
Total	39
Invalid	1
Total	40

	Statistical analysis
Mean	2.1
Std. Deviation	0.64
Minimum	1
Maximum	3

How would you rate the quality of the algorithm in supporting your decisions on the right treatment for myoma patients?	Frequency
Very high	1
High	20
Average	14
Low	3
Very low	0
Total	38
Invalid	2
Total	40

	Statistical analysis
Mean	2.5
Std. Deviation	0.69
Minimum	1
Maximum	4

Did you find the algorithm comprehensive in terms of the various aspects of fibroid treatment?	Frequency
Very comprehensive	1
Comprehensive	14
Partially comprehensive	20
Not sufficiently comprehensive	4
Not comprehensive at all	0
Total	39
Invalid	1
Total	40

	Statistical analysis
Mean	2.69
Std. Deviation	0.69
Minimum	1
Maximum	4

Do you think the algorithm will help improve patient outcomes by providing personalized treatment options?	Frequency
Definitively yes	2
Probably yes	17
Uncertain	12
Probably no	7
Definitively no	1
Total	39
Invalid	1
Total	40

	Statistical analysis
Mean	2.69
Std. Deviation	0.92
Minimum	1
Maximum	5

How likely is it that you will integrate this algorithm into your clinical routine for the treatment of fibroid patients?	Frequency
Very likely	1
Likely	14
Neutral	18
Unlikely	5
Very unlikely	1
Total	39
Invalid	1
Total	40

	Statistical analysis
Mean	2.77
Std. Deviation	0.81
Minimum	1
Maximum	5

Would you recommend this algorithm to your colleagues for the treatment of myoma patients?	Frequency
Definitively yes	4
Probably yes	13
Uncertain	18
Probably no	3
Definitively no	1
Total	39
Invalid	1
Total	40

	Statistical analysis
Mean	2.59
Std. Deviation	0.88
Minimum	1
Maximum	5

Do you think that using this algorithm could lead to a more efficient use of healthcare resources in fibroid treatment?	Frequency
I totally agree	6
I agree	18
Neutral	14
I disagree	1
I totally disagree	0
Total	39
Invalid	1
Total	40

	Statistical analysis
Mean	2.26
Std. Deviation	0.75
Minimum	1
Maximum	4

Overall, how satisfied are you with the user‐friendliness and quality of the AI‐generated myoma treatment algorithm?	Frequency
Very satisfied	2
Satisfied	24
Neutral	10
Unsatisfied	3
Very unsatisfied	0
Total	39
Invalid	1
Total	40

	Statistical analysis
Mean	2.36
Std. Deviation	0.71
Minimum	1
Maximum	4

On a scale of one to ten, how familiar are you with the use of artificial intelligence in medicine?	Statistical analysis
Mean	4.32
Std. Deviation	1.97
Minimum	1
Maximum	9

### 3.2 Patients' responses

All patients presented to the Department of Gynecology and Obstetrics, University Hospital Erlangen, Germany, for consultation regarding their fibroid burden. Twenty‐five patients had regular menses, while seven had experienced menopause. Nineteen patients had already received at least one previous therapeutic approach for their fibroids. Most patients were (somewhat) familiar with fibroids and their treatment options, found the algorithm easy (or at least neutral) to understand and assessed this tool as (somewhat) helpful in deciding on the right treatment for their fibroids. The majority of the patients felt that the algorithm at least largely adequately covered the different treatment options for fibroids and would at least probably consider using this algorithm to find the best treatment option for their fibroids. Twenty‐five patients believed that the use of this algorithm could probably empower them to have more informed conversations with their healthcare provider about fibroid treatment and were confident in the accuracy and reliability of the treatment suggestions provided by the algorithm. Most patients (64.4%) answered that this algorithm could help them better understand the treatment options and make decisions that matched their preferences and goals, whereas only five patients were unsatisfied with the user‐friendliness and helpfulness of the AI‐generated algorithm for fibroid treatment. The questions *How helpful do you think this algorithm would be for you in deciding on the right treatment for your fibroids?*, *Do you think using this algorithm could empower you to have more informed conversations with your healthcare provider about fibroid treatment?* and *Would you consider using this algorithm to find the best treatment option for your fibroids?* were the ones to receive the highest percentage of positive replies by the patients who were questioned, whereas the questions *Did you find the algorithm easy to understand and follow?* and *Overall, how satisfied are you with the user‐friendliness and helpfulness of the AI‐generated algorithm for fibroid treatment?* were the ones to receive the highest percentage of negative replies by the asked patient population (Table 2). Interestingly, patients who were familiar with fibroids and their treatment options more frequently answered that the algorithm promotes better understanding of the treatment options, thus helping them to make decisions that match their preferences and goals compared with those who are not so familiar with fibroids (Fisher exact test, 76.3% versus 0%, *P* = 0.006). The overall satisfaction, however, and evaluation of the algorithm did not differ among patients with diferrent levels of experience regarding fibroids (Fisher exact test, *P* < 0.10 for all associations).

**TABLE 2:** Patients‘ responses to the study questions regarding the algorithm.

How familiar are you with fibroids and their treatment options?	Frequency
Very familiar	3
Familiar	17
Somewhat familiar	19
Not very familiar	3
Not familiar at all	3
Total	45
Invalid	0
Total	45

	Statistical analysis
Mean	2.69
Std. Deviation	0.95
Minimum	1
Maximum	5

Did you find the algorithm easy to understand and follow?	Frequency
Very easy	5
Easy	11
Neutral	12
Difficult	7
Very difficult	1
Total	36
Invalid	9
Total	45

	Statistical analysis
Mean	2.67
Std. Deviation	1.04
Minimum	1
Maximum	5

How helpful do you think this algorithm would be for you in deciding on the right treatment for your fibroids?	Frequency
Very helpful	4
Helpful	26
Somewhat helpful	10
Not very helpful	1
Not helpful at all	1
Total	42
Invalid	3
Total	45

	Statistical analysis
Mean	2.26
Std. Deviation	0.77
Minimum	1
Maximum	5

Do you think that the algorithm adequately covers the different treatment options for fibroids?	Frequency
Fully adequately	4
Largely adequately	24
Partially adequately	11
Minimally adequately	1
Not adequately at all	0
Total	40
Invalid	5
Total	45

	Statistical analysis
Mean	2.23
Std. Deviation	0.66
Minimum	1
Maximum	4

Would you consider using this algorithm to find the best treatment option for your fibroids?	Frequency
Definitively yes	4
Propably yes	26
Neutral	11
Probably no	1
Definitively no	1
Total	43
Invalid	2
Total	45

	Statistical analysis
Mean	2.28
Std. Deviation	0.77
Minimum	1
Maximum	5

Do you think using this algorithm could empower you to have more informed conversations with your healthcare provider about fibroid treatment?	Frequency
Definitively yes	6
Propably yes	25
Neutral	9
Propably no	2
Definitively no	1
Total	43
Invalid	2
Total	45

	Statistical analysis
Mean	2.23
Std. Deviation	0.84
Minimum	1
Maximum	5

How confident are you in the accuracy and reliability of the treatment suggestions provided by the algorithm?	Frequency
Very confident	1
Confident	25
Neutral	12
Not very confident	4
Not confident at all	0
Total	42
Invalid	3
Total	45

	Statistical analysis
Mean	2.45
Std. Deviation	0.71
Minimum	1
Maximum	4

Do you think that using this algorithm could help you better understand your treatment options and make decisions that match your preferences and goals?	Frequency
Totally agree	6
Agree	23
Neutral	11
Disagree	2
Totally disagree	0
Total	42
Invalid	3
Total	45

	Statistical analysis
Mean	2.21
Std. Deviation	0.75
Minimum	1
Maximum	4

Overall, how satisfied are you with the user‐friendliness and helpfulness of the AI‐generated algorithm for fibroid treatment?	Frequency
Very satisfied	1
Satisfied	15
Neutral	14
Unsatisfied	5
Very unsatisfied	0
Total	35
Invalid	10
Total	45

	Statistical analysis
Mean	2.66
Std. Deviation	0.76
Minimum	1
Maximum	4

**FIGURE 1:**
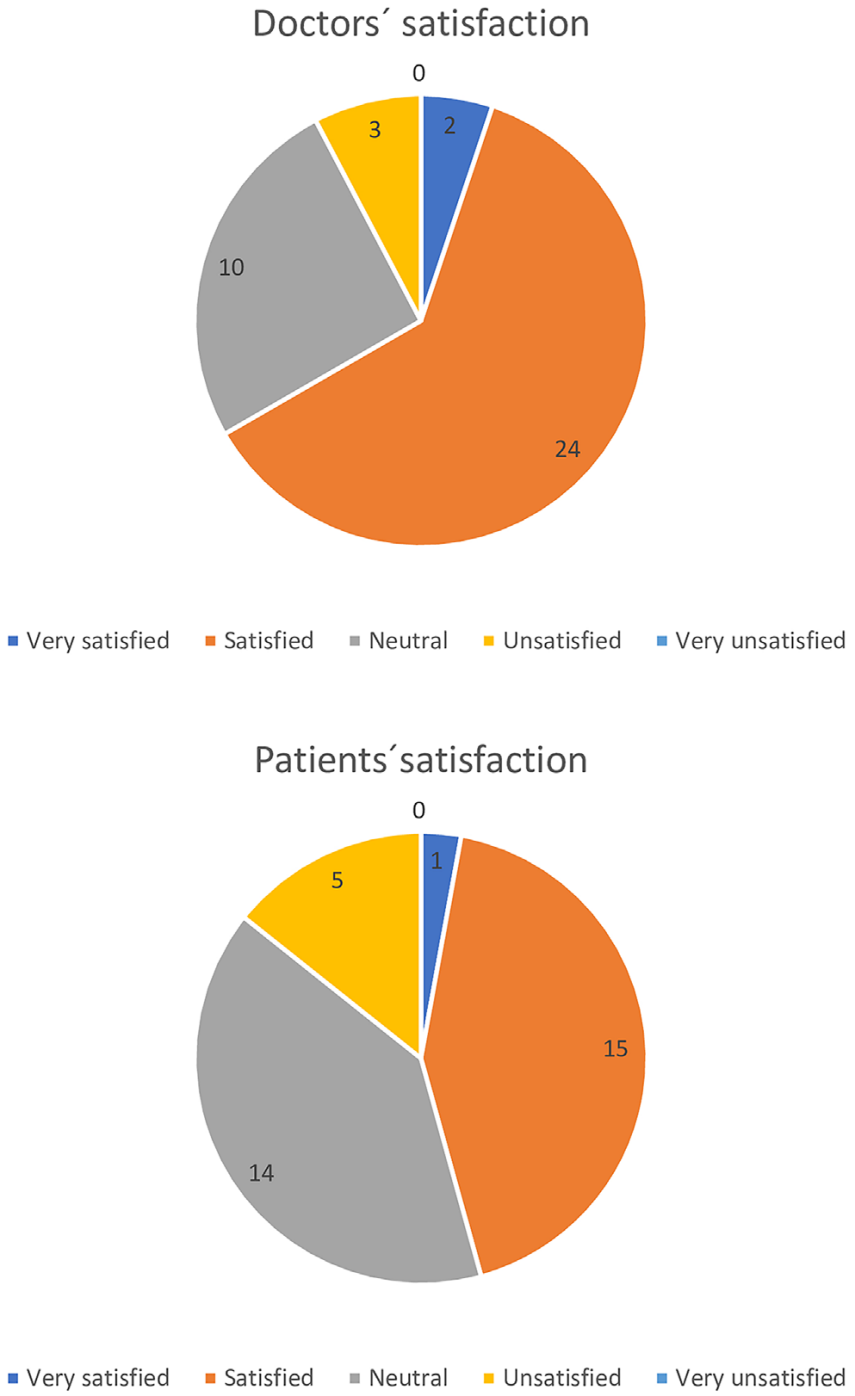
Doctors' and patients' satisfaction with the user‐friendliness and helpfulness of the AI‐generated algorithm for fibroid treatment.

Figure 1 graphically depicts both the physicians' and the patients' satisfaction with the user‐friendliness and helpfulness of the AI‐generated algorithm for fibroid treatment.

### 3.3 Comparison of the results between clinicians and patients

In our questionnaires, clinicians found the algorithm much easier (Fisher exact test, 74.4% versus 44.4%, *P* = 0.008) and tended to be overall more satisfied with the user‐friendliness and helpfulness of the AI‐generated algorithm for fibroid treatment (Fisher exact test, 66.7% versus 45.7%, *P* = 0.069) compared with patients; the latter association, however, being of marginal significance. On the other hand, patients evaluated the algorithm more often as comprehensive compared with clinicians, in terms of covering the various treatment options of fibroid treatment (Fisher exact test, 70% versus 38.4%, *P* = 0.005) and displayed a higher tendency of considering using the algorithm for this purpose (Fisher exact test, 69.7% versus 38.5%, *P* = 0.003).

## 4 DISCUSSION

The management of uterine fibroids presents a complex clinical scenario characterized by diverse symptomatology and therapeutic considerations. Our study aimed to address the unmet need for personalized treatment approaches in Germany by leveraging AI to generate feasible evidence‐based recommendations tailored to individual patient profiles. Through the development of an accessory algorithm using ChatGPT, we sought to create a supportive tool for the clinical setting that would empower both patients and clinicians with potential personalized treatment plans that account for the heterogeneity of fibroid presentations and patient preferences.

Most physicians, familiar with AI in medicine, found the algorithm user‐friendly and comprehensive for fibroid treatment, though only half believed it would improve patient outcomes. Despite this fact, 15 physicians indicated a willingness to integrate the algorithm into practice, and 24 felt it could enhance healthcare efficiency. Patients generally found the algorithm easy to understand and helpful in exploring treatment options, with 25 stating it could empower informed discussions with their providers. Most patients felt the algorithm adequately covered therapeutic choices and aligned with their preferences, while only a minority expressed dissatisfaction with its usability or helpfulness.

Artificial intelligence has emerged as a transformative tool in gynecology and obstetrics, with studies demonstrating its efficacy across various applications. In the domain of prenatal care, AI algorithms have shown remarkable accuracy in fetal anomaly detection through advanced image analysis in ultrasonography, outperforming traditional diagnostic methods in some cases.^18^ Additionally, predictive models leveraging machine learning have been employed to assess risks for complications such as pre‐eclampsia, preterm birth, and gestational diabetes, enabling timely and personalized interventions.^19^ In gynecology, AI‐driven tools are for instance being used for early detection of ovarian cancers through improved imaging interpretation and biomarker analysis.^20^ Robotic‐assisted surgeries, guided by AI, are enhancing precision and reducing recovery times, significantly benefiting patients.^21^ These advancements underscore the potential of AI to improve diagnostic accuracy, optimize treatment protocols, and enhance overall patient outcomes, positioning it as a supportive component of modern obstetric and gynecologic practice.

Our findings underscore the necessity of providing tailor‐made treatment plans for patients with uterine fibroids, reflecting the inherent variability in symptom severity, fibroid location, and patient goals. By synthesizing information from international suggestions and local expertise,^15^, ^16^, ^17^ our algorithm aimed to bridge the gap between available evidence and clinical decision making in the absence of an official national guideline in Germany. The individualized nature of our approach aligns with the principles of patient‐centered care, prioritizing the unique needs and preferences of each patient.

It is important to acknowledge that while our algorithm offers valuable insights and recommendations, it cannot replace the role of formal guidelines in guiding clinical practice. The development and dissemination of official guidelines by the German Society for Gynecology and Obstetrics are essential to standardize care and ensure consistency in treatment approaches across healthcare settings. A unified statement from the professional body will provide clinicians with a standardized framework for managing uterine fibroids, incorporating the latest evidence‐based recommendations and expert consensus.

Nevertheless, our algorithm represents a valuable tool for daily clinical practice once official guidelines are established. By adapting our algorithm to align with the recommendations outlined in the forthcoming guideline, we can facilitate seamless integration into routine clinical workflows. The algorithm can serve as a decision support tool for clinicians, offering real‐time guidance based on patient‐specific parameters and the latest evidence‐based practices. Additionally, it can enhance patient education and engagement by providing transparent information about treatment options and expected outcomes.

The inclusion of both perspectives aims at highlighting the potential for ChatGPT to act as a supportive tool, aiding in communication and providing relevant information for patients and healthcare providers. Even though ChatGPT cannot replace the need for formal, evidence‐based clinical guidelines, it can serve as an initial step toward standardizing treatment protocols and offering more personalized insights. It may indeed play a role in assisting gynecologic societies with drafting and updating official treatment policies.

However, a few limitations should be considered, with a special focus on the single‐center nature of the study and the small sample size. First, ChatGPT's responses are based on the data it has been trained on, and may not always incorporate the latest research or individual patient nuances. Although it can offer general guidance, it cannot replace the clinical judgment or hands‐on expertise of trained professionals. Moreover, the lack of personalized medical advice remains a significant concern, as the tool may not fully account for the complexities of each patient's specific case, such as comorbidities or unique treatment needs. Additionally, while ChatGPT's accessibility and convenience are advantages, there is a risk of oversimplification in the information it provides. For example, the recommendation of a treatment plan based on general knowledge may not always align with the current evidence or be suited for the patient's context. Hence, its use should be accompanied by careful review from a healthcare provider. Finally, rigorous validation in clinical settings is essential for ChatGPT to be widely adopted. It will need to undergo more extensive testing, including feedback loops from real‐world clinical scenarios, before it can be considered a reliable and accurate tool in fibroid management. This process would also include addressing ethical concerns and ensuring patient privacy is not compromised in any way.

In conclusion, our study highlights the importance of personalized treatment planning in uterine fibroid therapy and underscores the potential of AI‐driven solutions to support clinical decision making in the absence of formal guidelines. Our algorithm cannot replace the need for official guidelines, but it represents a promising adjunctive tool that can enhance the quality and efficiency of care for patients with uterine fibroids in Germany. Moving forward, collaboration between clinicians, professional societies, and AI developers will be essential to ensure the effective integration of AI technologies into clinical practice and optimize patient outcomes.

## AUTHOR CONTRIBUTIONS

IP and JE contributed to writing—conceptualization and original draft preparation; LL, NS, FH, GL, and JE contributed to review and editing, formal analysis, and supervision. All authors have read and agreed to the published version of the manuscript.

## CONFLICT OF INTEREST STATEMENT

The authors have no conflicts of interest.

## Supporting information


Data S1.

Data S2.

## Data Availability

Research data are not shared.
