# Supplementary material for: Leveraging artificial intelligence for evidence‐based recommendations in uterine fibroid therapy: Addressing the unmet need in German healthcare—A clinical trial
Source: Int J Gynaecol Obstet. 2025 Jul 29;172(2):1104–13. doi: 10.1002/ijgo.70407 (PMC12790660; doi:10.1002/ijgo.70407)
Supplement: Supplementary file 1 — Data S1. [file IJGO-172-1104-s002.docx]

**Algorithmus**

*Wir beauftragten Chat-GPT (ein Künstliche-Intelligenz-Programm) mit der Synthese von Informationen aus der internationalen und deutschen medizinischen Literatur, um evidenzbasierte Empfehlungen für die Therapie von Uterusmyomen zu erstellen. Der von ChatGPT entwickelte Algorithmus zielte darauf ab, die bestgeeigneten therapeutischen Optionen für einzelne Patientinnen auf der Grundlage ihrer klinischen Merkmale, ihrer Präferenzen und der in den bereitgestellten Informationen dargelegten Zulassungskriterien zu ermitteln.*

**Teil 1: Einstimmiger Vorschlag für Patientinnen mit Myomen – Wovon hängt zunächst die Entscheidung ab, welche Therapie für jede Patientin in Frage kommen könnte?**

1. **Kinderwunsch:** Berücksichtigung, ob die Patientin eine Fertilitätserhaltung wünscht oder die Familienplanung abgeschlossen hat.
2. **Symptomatik-Schweregrad:** Klinische Bewertung der Schwere der Symptome wie starke Menstruationsblutungen, Beckenschmerzen oder Druckbeschwerden.
3. **Myom-Eigenschaften:** Bewertung von Größe, Lage und Anzahl der Myome, um den am besten geeigneten Behandlungsansatz zu bestimmen.
4. **Patientenpräferenzen:** Besprechung mit der Patientin über ihre Behandlungswünsche, einschließlich der Komfortzone bei verschiedenen operativen und nicht- operativen Optionen.
5. **Gesundheitszustand:** Berücksichtigung des allgemeinen Gesundheitszustands der Patientin, bestehender Begleiterkrankungen und Eignung für verschiedene Behandlungsmethoden.

*Unter Berücksichtigung dieser Faktoren wäre die einstimmige Empfehlung für Patientinnen mit Myomen, eine gemeinsame Entscheidungsfindung mit ihrem Arzt zu treffen, um einen personalisierten Behandlungsplan zu erstellen.*

Dieser Plan kann Folgendes umfassen:

**Konservatives (Abwartendes) Management:** Für asymptomatische Patientinnen oder solche mit leichten Symptomen kann eine erwartungsvolle Behandlung mit regelmäßiger Überwachung angemessen sein.

**Medizinische/interventionelle Therapie:** Für symptomatische Patientinnen, die keine Operation wünschen, können medizinische Therapien wie hormonelle Medikamente (z.B. GnRH-Modulatoren) oder eine Uterusarterienembolisation bzw. Radiofrequenzablation in Betracht gezogen werden.

**Chirurgische Intervention:** Für Patientinnen mit schweren Symptomen oder solche, die eine Fertilitätserhaltung wünschen, können je nach individuellen Umständen der Patientin chirurgische Optionen wie eine Myomektomie (hysteroskopisch, laparoskopisch oder abdominal) oder die Hysterektomie empfohlen werden.

**Teil 2: Ein-/Ausschlusskriterien für die unterschiedlichen Myom-Behandlungsoptionen**

**Hysteroskopische Myomektomie:**

*Geeignete Kandidaten:*

Patientinnen mit submukösen Myomen, die starke Menstruationsblutungen verursachen.

Diejenigen, die eine Fertilitätserhaltung wünschen, insbesondere wenn die Myome hauptsächlich submukös sind.

*Ausgeschlossene Kandidaten:*

Patientinnen mit großen oder intramuralen Myomen, die nicht für eine hysteroskopische Resektion geeignet sind.

Diejenigen mit Myomen, die überwiegend außerhalb der Gebärmutterhöhle liegen.

**Laparoskopische oder abdominale Myomektomie:**

*Geeignete Kandidaten:*

Patientinnen mit mehreren oder größeren Myomen, einschließlich intramuraler und subseröser Myome.

Diejenigen, die eine Fertilitätserhaltung wünschen, aber nicht für eine hysteroskopische Myomektomie geeignet sind.

*Ausgeschlossene Kandidaten:*

Patientinnen mit umfangreicher Myom-Belastung oder schlechter chirurgischer Eignung aufgrund von Begleiterkrankungen.

Diejenigen mit erheblichen Verwachsungen oder anderen intraabdominalen Pathologien, die die Operation erschweren.

**Uterusarterienembolisation (UAE):**

*Geeignete Kandidaten:*

Patientinnen mit mehreren oder großen Myomen, die nicht für eine chirurgische Intervention geeignet sind.

Diejenigen mit symptomatischen Myomen, die eine weniger invasive Behandlungsoption suchen.

*Ausgeschlossene Kandidaten:*

Patientinnen, die eine Fertilitätserhaltung wünschen, da UAE die Eierstockfunktion beeinträchtigen kann.

Diejenigen mit Myomen, die eine erhebliche Verformung der Gebärmutterhöhle verursachen oder die Fruchtbarkeitspotenziale beeinträchtigen.

**Erwartungsvolles Management:**

*Geeignete Kandidaten:*

Asymptomatische Patientinnen mit kleinen, stabilen Myomen.

Diejenigen, die versuchen, schwanger zu werden, ohne signifikante Symptome.

*Ausgeschlossene Kandidaten:*

Patientinnen mit symptomatischen Myomen, die die Lebensqualität oder das Fruchtbarkeitspotenzial beeinträchtigen.

Diejenigen mit schnell wachsenden oder symptomatischen Myomen, die eine Intervention erfordern.

**Medikamentöse-Hormonelle Therapie:**

*Geeignete Kandidaten:*

Patientinnen mit leichten bis mäßigen Symptomen, die keine chirurgische Intervention wünschen.

Diejenigen mit Kontraindikationen für eine Operation oder die nicht-invasive Optionen bevorzugen.

*Ausgeschlossene Kandidaten:*

Patientinnen mit schweren Symptomen, die nicht auf medikamentöse Therapie ansprechen.

Diejenigen mit Kontraindikationen für die spezifischen Medikamente zur Myom-Behandlung.

**Teil 3: Erst- und Zweitlinientherapien für die Myom-Patientinnen**

**Erstlinientherapie:**

*Für Patientinnen, die eine Fertilitätserhaltung wünschen:*

Nur submuköse Myome:

Hysteroskopische Myomektomie: In Betracht ziehen bei symptomatischen submukösen Myomen, die starke Menstruationsblutungen verursachen.

Alle anderen Myome außer submukösen:

Laparoskopische Myomektomie: Bevorzugt für Patientinnen mit einer Uterusgröße <17 Wochen und weniger als fünf Myome.

*Für Patientinnen, die keine Fertilitätserhaltung wünschen:*

Symptomatische Patientinnen:

Chirurgische Myomektomie (laparoskopisch oder abdominal): In Betracht ziehen bei Druckbeschwerden oder Myomen, die nicht für eine hysteroskopische Resektion geeignet sind.

**Zweitlinientherapie (wenn die Erstlinientherapie nicht möglich oder unwirksam ist):**

*Für Patientinnen, die eine Fertilitätserhaltung wünschen:*

Nur submuköse Myome:

Laparoskopische Myomektomie: In Betracht ziehen, wenn die hysteroskopische Myomektomie nicht möglich oder erfolglos ist.

Alle anderen Myome außer submukösen:

Offene abdominale Myomektomie: In Betracht ziehen bei Patientinnen mit zahlreichen oder größeren Myomen, die nicht für einen laparoskopischen Eingriff geeignet sind.

*Für Patientinnen, die keine Fertilitätserhaltung wünschen:*

Symptomatische Patientinnen:

Uterusarterienembolisation (UAE): In Betracht ziehen bei Patientinnen mit Kontraindikationen für eine Operation oder Präferenz für eine minimalinvasive Option.

**Fortlaufende Überwachung und Bewertung:**

Patientinnen regelmäßig auf Symptomrückkehr oder Myomwachstum überwachen.

Behandlungsmodalitäten bei Änderungen des klinischen Status der Patientin oder der Behandlungsziele neu bewerten.
