# Supplementary material for: Leveraging artificial intelligence for evidence‐based recommendations in uterine fibroid therapy: Addressing the unmet need in German healthcare—A clinical trial
Source: Int J Gynaecol Obstet. 2025 Jul 29;172(2):1104–13. doi: 10.1002/ijgo.70407 (PMC12790660; doi:10.1002/ijgo.70407)
Supplement: Supplementary file 2 — Data S2. [file IJGO-172-1104-s001.docx]

**Fragebogen für Ärzte**

Datum:

1. Wie häufig sehen Sie Patientinnen mit Myomen in Ihrem klinischen Alltag?

a) Sehr häufig

b) Häufig

c) Gelegentlich

d) Selten

e) Nie

*Bitte geben die Anzahl von Patientinnen mit Myomen, die Sie jährlich behandeln:*

2. Hatten Sie die Gelegenheit, den KI-erstellten Algorithmus zur Behandlung von Myomen zu überprüfen?

a) Ja, ausführlich

b) Ja, kurz

c) Nein, noch nicht

d) Nein, ich bin nicht interessiert

e) Nicht zutreffend

3. Finden Sie den Algorithmus leicht zu verstehen und in Ihrem klinischen Alltag einsetzbar?

a) Sehr einfach

b) Einfach

c) Neutral

d) Schwierig

e) Sehr schwierig

4. Wie würden Sie die Qualität des Algorithmus bei der Unterstützung Ihrer Entscheidungen zur richtigen Behandlung von Myom-Patientinnen bewerten?

a) Ausgezeichnet

b) Gut

c) Durchschnittlich

d) Schlecht

e) Sehr schlecht

5. Haben Sie den Algorithmus als umfassend in Bezug auf die verschiedenen Aspekte der Myom-Behandlung empfunden?

a) Äußerst umfassend

b) Umfassend

c) Etwas umfassend

d) Nicht sehr umfassend

e) Überhaupt nicht umfassend

6. Glauben Sie, dass der Algorithmus dazu beiträgt, die Patientenergebnisse zu verbessern, indem er personalisierte Behandlungsoptionen bietet?

a) Definitiv

b) Wahrscheinlich

c) Unsicher

d) Wahrscheinlich nicht

e) Definitiv nicht

7. Wie wahrscheinlich ist es, dass Sie diesen Algorithmus in Ihre klinische Routine zur Behandlung von Myom-Patientinnen integrieren?

a) Sehr wahrscheinlich

b) Wahrscheinlich

c) Neutral

d) Unwahrscheinlich

e) Sehr unwahrscheinlich

8. Würden Sie diesen Algorithmus Ihren Kollegen zur Behandlung von Myom-Patientinnen empfehlen?

a) Definitiv

b) Wahrscheinlich

c) Unsicher

d) Wahrscheinlich nicht

e) Definitiv nicht

9. Glauben Sie, dass die Verwendung dieses Algorithmus zu einer effizienteren Nutzung von Gesundheitsressourcen bei der Myom-Behandlung führen könnte?

a) Stimme voll zu

b) Zustimmen

c) Neutral

d) Ablehnen

e) Stimme überhaupt nicht zu

10. Insgesamt, wie zufrieden sind Sie mit der Benutzerfreundlichkeit und Qualität des KI-erstellten Algorithmus zur Myom-Behandlung?

a) Sehr zufrieden

b) Zufrieden

c) Neutral

d) Unzufrieden

e) Sehr unzufrieden

11. Was ist Ihr Fachgebiet/Spezialisierung?

a) Gynäkologie (Klinik)

b) Gynäkologie (Praxis)

c) Allgemeinmedizin

d) Interventionelle Radiologie

12. Welche Behandlungsmethoden bevorzugen/schlagen Sie für Myom-Patientinnen vor? Und warum?

a) Abwartendes Vorgehen

b) Konservative medikamentöse Behandlung (bitte angeben)

c) Chirurgische Behandlung (hysteroskopisch; laparoskopisch; laparotomisch)

d) Minimalinvasive Alternativen zur Operation (Embolisation; fokussierter Ultraschall; Ablation)

Gründe: (Freitext)

13. Gab es in dem Algorithmus Behandlungsoptionen, von denen Sie nichts wussten? Fehlen Ihnen Behandlungsoptionen, die im Algorithmus nicht abgedeckt sind?

a) Abwartendes Vorgehen

b) Konservative medikamentöse Behandlung (bitte angeben)

c) Chirurgische Behandlung (hysteroskopisch; laparoskopisch; laparotomisch)

d) Minimalinvasive Alternativen zur Operation (Embolisation; fokussierter Ultraschall; Ablation)

e) Andere: bitte angeben

14. Basierend auf Ihrer Erfahrung auf dem Gebiet der Myom-Behandlung, welche Therapieoptionen sind Ihrer Meinung nach am besten für Myome geeignet?

a) Abwartendes Vorgehen

b) Konservative medikamentöse Behandlung (bitte angeben)

c) Chirurgische Behandlung (hysteroskopisch; laparoskopisch; laparotomisch)

d) Minimalinvasive Alternativen zur Operation (Embolisation; fokussierter Ultraschall; Ablation)

e) Andere: bitte angeben

15. Auf einer Skala von eins bis zehn, wie vertraut sind Sie mit der Verwendung von künstlicher Intelligenz in der Medizin?

16. Kennen Sie die Richtlinie über die Konkretisierung des Anspruchs auf eine unabhängige ärztliche Zweitmeinung?

*Die Richtlinie bestimmt, für welche planbaren Eingriffe Patientinnen und Patienten einen Rechtsanspruch auf eine unabhängige ärztliche Zweitmeinung haben. Sie legt außerdem die allgemeinen und indikationsspezifischen Anforderungen an das Zweitmeinungsverfahren und an die Erbringer einer Zweitmeinung fest.*

a) Ja, und zwar: (Informationsquelle)

b) Nein

**Fragebogen für Myom-Patientinnen:**

Datum:

1. Wie vertraut sind Sie mit Myomen und deren Behandlungsmöglichkeiten?

a) Sehr vertraut

b) Vertraut

c) Etwas vertraut

d) Nicht sehr vertraut

e) Überhaupt nicht vertraut

2. Hatten Sie die Gelegenheit, den KI-erstellten Algorithmus zur Myom-Behandlung zu überprüfen?

a) Ja, ausführlich

b) Ja, kurz

c) Nein, noch nicht

d) Nein, ich bin nicht interessiert

e) Nicht zutreffend

3. Fanden Sie den Algorithmus leicht zu verstehen und zu befolgen?

a) Sehr einfach

b) Einfach

c) Neutral

d) Schwierig

e) Sehr schwierig

4. Wie hilfreich glauben Sie, dass dieser Algorithmus für Sie bei der Entscheidung über die richtige Behandlung Ihrer Myome sein würde?

a) Äußerst hilfreich

b) Hilfreich

c) Etwas hilfreich

d) Nicht sehr hilfreich

e) Überhaupt nicht hilfreich

5. Sind Sie der Meinung, dass der Algorithmus die verschiedenen Behandlungsoptionen für Myome angemessen abdeckt?

a) Vollständig abgedeckt

b) Großteils abgedeckt

c) Teilweise abgedeckt

d) Minimale Abdeckung

e) Überhaupt nicht abgedeckt

6. Würden Sie erwägen, diesen Algorithmus zu verwenden, um die beste Behandlungsoption für Ihre Myome zu finden?

a) Definitiv

b) Wahrscheinlich

c) Unsicher

d) Wahrscheinlich nicht

e) Definitiv nicht

7. Glauben Sie, dass die Verwendung dieses Algorithmus Sie dazu befähigen könnte, informiertere Gespräche mit Ihrem Gesundheitsdienstleister über die Myom-Behandlung zu führen?

a) Definitiv

b) Wahrscheinlich

c) Unsicher

d) Wahrscheinlich nicht

e) Definitiv nicht

8. Wie zuversichtlich sind Sie in die Genauigkeit und Zuverlässigkeit der Behandlungsvorschläge, die der Algorithmus liefert?

a) Sehr zuversichtlich

b) Zuversichtlich

c) Neutral

d) Nicht sehr zuversichtlich

e) Überhaupt nicht zuversichtlich

9. Glauben Sie, dass die Verwendung dieses Algorithmus Ihnen helfen könnte, Ihre Behandlungsoptionen besser zu verstehen und Entscheidungen zu treffen, die Ihren Vorlieben und Zielen entsprechen?

a) Stimme voll zu

b) Zustimmen

c) Neutral

d) Ablehnen

e) Stimme überhaupt nicht zu

10. Insgesamt, wie zufrieden sind Sie mit der Benutzerfreundlichkeit und der Hilfreichkeit des KI-erstellten Algorithmus zur Myom-Behandlung?

a) Sehr zufrieden

b) Zufrieden

c) Neutral

d) Unzufrieden

e) Sehr unzufrieden

11. Falls Sie zuvor Behandlungen für Ihre Myome hatten, geben Sie bitte an, wann und welche Art der Behandlung:

a) Abwartendes Vorgehen

b) Konservative medikamentöse Behandlung (bitte angeben)

c) Chirurgische Behandlung (hysteroskopisch; laparoskopisch; laparotomisch)

d) Minimalinvasive Alternativen zur Operation (Embolisation; fokussierter Ultraschall; Ablation)

e) Andere: bitte angeben

12. Kennen Sie die Richtlinie über die Konkretisierung des Anspruchs auf eine unabhängige ärztliche Zweitmeinung?

*Die Richtlinie bestimmt, für welche planbaren Eingriffe Patientinnen und Patienten einen Rechtsanspruch auf eine unabhängige ärztliche Zweitmeinung haben. Sie legt außerdem die allgemeinen und indikationsspezifischen Anforderungen an das Zweitmeinungsverfahren und an die Erbringer einer Zweitmeinung fest.*

a) Ja, und zwar: (Informationsquelle)

b) Nein

13. Stellen Sie sich aktuell zur Zweitmeinung vor?

a) Ja

b) Nein

14. Haben Sie noch Ihre Regelblutung?

a) Ja, regelmäßig

b) Ja, allerdings unregelmäßig (Wechseljahre)

c) Nein, seit über einem Jahr nicht mehr (Menopause)
